# Utility of routine preoperative laboratory testing for patients undergoing minor gynaecologic surgical procedures: interim analysis of their impact on intraoperative and postoperative complications

**DOI:** 10.52054/FVVO.16.3.027

**Published:** 2024-09-30

**Authors:** U Catena, A Biasioli, C Paglietti, V Tarantino, G Pellecchia, G Esposito, F Previtera, S Zermano, M Arcieri, A Graziano, G Dinoi, F Ciano, L Driul, G Draisci, F Fanfani, G Scambia, G Vizzielli, S Restaino

**Affiliations:** UOC Ginecologia Oncologica, Dipartimento per la salute della Donna e del Bambino e della Sanità Pubblica, Fondazione Policlinico Universitario A. Gemelli, IRCCS, Largo Agostino Gemelli, 00168, Rome, Italy; Department of Maternal and Child Health, “Santa Maria della Misericordia” University Hospital, Azienda Sanitaria Universitaria Friuli Centrale (ASUFC), Piazzale della Misericordia, 33100, Udine, Italy; Department of Medicine (DMED), University of Udine, 33100 Udine, Italy; Università Cattolica del Sacro Cuore, Largo Francesco Vito, 00168, Rome, Italy; Department of Biomedical, Dental, Morphological and Functional Imaging Science, University of Messina, 98122 Messina, Italy; Department of Anaesthesiology and Resuscitation Medicine, Sacro Cuore Catholic University, Largo Francesco Vito, 00168, Rome, Italy

**Keywords:** Gynaecological surgery, hysteroscopy, laboratory, preoperative, blood test, surgical complications

## Abstract

**Background:**

Despite discouragement from many scientific societies, routine preoperative testing remains a common practice. Minor gynaecological surgery, being widely performed in everyday practice, represents an opportunity for implementing cost-reduction policies by avoiding unnecessary diagnostic assessments.

**Objectives:**

To assess whether performing routine preoperative blood tests affects postoperative complications and cost-effectiveness in patients undergoing minor gynaecological surgery.

**Materials and Methods:**

An interim subgroup analysis of a retrospective study conducted by Fondazione Policlinico Gemelli (Rome) and Azienda Sanitaria Universitaria Friuli Centrale (Udine) was performed. Patients who underwent surgery under general anaesthesia were included. The studied population was divided based on the preoperative work up. Clinical data, surgical features and complications were collected.

**Main outcome measures:**

Intraoperative and postoperative complications, healthcare expenditure in two groups.

**Results:**

Subgroup analysis included 1191 patients in Centre A (Rome) who underwent routine complete preoperative tests and 500 patients in Centre B (Udine), who underwent exams only if indicated. Population characteristics were similar in two groups. Postoperative complications were observed in 1.2% and 1.4% of cases in Group A and Group B, respectively (p=0.70). Severe complications occurred in 0.3% of cases in Group A and 0.4% in Group B. Group B showed a cost saving of approximately 70 Euros per procedure (p < 0.001).

**Conclusions:**

Preliminary data indicate that routine perioperative assessment did not reduce complication rates, hospital readmissions or surgical reinterventions. Given the high number of procedures, performing specific preoperative tests only when indicated may result in significant cost reduction.

**What is new?:**

This study selectively highlights the potential benefits to overall public health expenditure that could be achieved through stricter adherence to guidelines on preoperative assessment in minor gynaecological surgery.

## Introduction

Minor gynaecological surgery encompasses various types of minimally invasive procedures, such as hysteroscopy, cervical loop electrode excision procedure (LEEP), conisation and removal of vulvar lesions. It is typically conducted in a day-case and often outpatient setting. Their widespread adoption has brought about a radical transformation in gynaecological clinical practice, as these procedures require minimal surgical time and can safely address a range of conditions (Carugno et al., 2022).

There is substantial evidence demonstrating a very low complication rate with minor gynaecological surgery. For example, a large multicentre study examining 13,600 procedures, reported complication rates of 0.13% for diagnostic hysteroscopies and 0.28% for operative hysteroscopies. Notably, half of the complications were attributed to the method of entry into the uterine cavity (e.g., cervical dilation), while the other half were linked to the surgeon’s expertise. Additionally, hysteroscopic intrauterine adhesiolysis was identified as the riskiest procedure, with a complication rate of 4.5% (Jansen et al., 2000). Similar findings were reported by Aydeniz et al. (2002) who identified uterine perforations as the most common complication (0.12%), followed by fluid overload syndrome (0.06%), bleeding (0.03%), and bladder or bowel injuries related to uterine perforation (0.02%), with infections occurring in 0.01% of cases. In many gynaecological facilities these procedures require a preoperative diagnostic work-up. This could help determine whether patients can safely undergo the intended intervention and might also screen those who require further perioperative optimisation to reduce complications and improve outcomes.

The preoperative workup typically involves history taking and assessment of previous medical records and physical and ultrasonographic examination. In some cases, it could also include haematological testing (full blood count, coagulation testing, urea and electrolytes and liver function tests), urinalysis, electrocardiography (ECG), and chest radiography. Additional specific investigations may be required based on anaesthetic considerations. The utility and appropriateness of routinely performing these investigations have been debated for decades. A landmark study published in JAMA in 1985 evaluated the utility of routine laboratory screening of preoperative patients. The study, which assessed the laboratory, demographic, and discharge diagnostic data of 2,000 patients undergoing elective surgery, found that 60% of the routinely administered lab tests would not have been performed if testing had only been conducted for recognisable indications (Kaplan et al., 1985). Many other studies have been published thereafter refuting the use of routine preoperative laboratory tests before minor surgical procedures. In 2002, The American Society of Anaesthesiologists (ASA) released the initial set of preoperative assessment guidelines, which are continually reviewed and updated. The latest version reaffirmed that “preoperative tests should not be ordered routinely in the absence of clinical indication” and outlined specific conditions in which these tests are required. These conditions include the type and invasiveness of the procedure, concurrent liver disease, extremes of age, and a history of anaemia, bleeding, or other haematological disorders. Although not specifically mentioned, minor gynaecological surgery is widely considered typical of minimally invasive surgical procedures. Therefore, routine preoperative tests should not be conducted, and the presence of other selected clinical characteristics should guide the anaesthetist (Committee on Standards and Practice Parameters, 2012). Subsequent guidelines from other scientific societies, such as The National Institute for Health and Care Excellence (NICE) in the United Kingdom, the European Society of Anaesthesiology, and the American Cardiology Association/American Heart Association (ACA/ AHA), closely align with those of the ASA (NICE, 2016; De Hert et al., 2018; Fleisher et al., 2014). Nevertheless, a recent study published in 2021 revealed that preoperative laboratory testing continues to be routinely performed for most low- risk patients undergoing outpatient gynaecologic surgery (Mutter et al., 2021).

This practice, despite updated evidence-based guidelines, represents a significant waste of resources. It is estimated that 18 billion dollars are annually spent on preoperative testing in the United States alone (Richman, 2010). In a healthcare context where institutions emphasise the importance of cost reduction and improved patient care, identifying the safest and most cost-effective management is imperative. Considering this background, our study aimed to investigate whether, in the absence of risk factors, preoperative blood tests resulted in different outcomes in terms of intraoperative and postoperative complications in patients undergoing minor gynaecological surgical procedures. We also analysed the cost-effectiveness of the two different preparatory workups.

## Methods

A retrospective observational study was conducted jointly by Fondazione Policlinico Universitario A. Gemelli IRCCS (Rome) and the Clinic of Obstetrics and Gynaecology of the Azienda Sanitaria Universitaria Friuli Centrale (Udine).

All patients who underwent minor gynaecologic surgery at CLASS Hysteroscopy centre in Policlinico Universitario A. Gemelli (Centre A) or at the Clinic of Obstetrics and Gynaecology of the Azienda Sanitaria Universitaria Friuli Centrale (Udine) (Centre B) between January 2017 and August 2023 were enrolled.

### Inclusion criteria:

- Patients >= 18 years old
- Patients who underwent minor gynaecologic surgery (operative or diagnostic hysteroscopy, uterine cavity revision, LEEP or conisation, vulvar procedures etc.)
- Patients treated under conscious sedation or general anaesthesia.

### Exclusion criteria:

- Office Hysteroscopic procedures
- Minor gynaecological surgical procedures combined with major procedures (e.g. hysteroscopy for uterine cavity evaluation during a laparoscopic/laparotomic procedure)
- Patients at high anaesthesiologic risk (American Society of Anaesthesiologist – ASA 3)
- Patients with a known swab positivity for SARS-Cov2
- Patients with missing data

For each patient the following data were collected: demographics, medical history including BMI, the American Society of Anaesthesiologist physical status classification system (ASA score), allergies, and comorbidities; surgical information such as the procedure performed, type of anaesthesia, operation time, post procedural complications (reported according to the Clavien-Dindo classification) and length of hospitalisation. All collected records were anonymised and patients with incomplete demographic or clinical data were excluded. The study population was divided into two groups: ‘Group A’ (Policlinico Gemelli, Rome), where all patients underwent routine preoperative blood tests (defined as full blood count, coagulation testing, urea and electrolytes and liver function tests obtained within 30 days before surgery) as well as chest X-rays, and ‘Group B’, where these assessments were performed only if indicated. In both groups, each patient underwent a thorough medical history assessment with a review of previous medical records, as well as a physical and ultrasonographic evaluation, an anaesthesiologic assessment and an electrocardiogram (ECG). The cost of each preoperative procedure was calculated according to regional schedule for health services. Prefacing that healthcare service fees in Italy vary from region to region, according to Regione Lazio tariff schedule, the cost of full blood count, glucose level, urea, electrolytes, liver function and haemostasis tests is 55 Euros for each patient, while the cost of a chest X-ray in two projections is 15 Euros. Given that the ECG is performed in both facilities, this cost is not included in the expenses. Clinical, demographic, surgical and postprocedural characteristics were compared between the two groups. Data are reported as median or percentage for each group. Descriptive statistics were used to characterise the patient population and the surgical features. Because of the non-randomised nature of the study design and the potential allocation biases arising from the retrospective comparison between the two groups, we performed propensity score matching. A propensity score was developed through a multivariate logistic regression model. The sample size after matching was 7059 (N cases = 2353 and N controls = 4706). This dimension allowed us to detect, with a power of 80%, an expected proportion of complications of 0.30% in the exposed group and of 0.85% in the control group, with two-sided alpha = 0.05. The chi- square test and Fisher exact test was used to compare variables. Differences were considered statistically significant if p < 0.05. Institutional Review Board approval was obtained.

## Results

Of the total cohort of 7059 patients, a subgroup of 1691 were used for this interim analysis: 1191 patients treated at CLASS Hysteroscopy Centre in Fondazione Policlinico Universitario A. Gemelli IRCCS, and 500 patients treated at Clinic of Azienda Sanitaria Universitaria Friuli Centrale (Udine). A breakdown of the procedures performed in each centre is presented in Table I. Patients from both centres underwent medical history collection, as well as physical and ultrasonographic evaluations, ECG and anaesthetic assessment. Each patient in Centre A (Rome) was offered a routine assessment including full blood count, haemostasis, urea and electrolyte levels, liver function tests and chest X-rays. Conversely, in Centre B (Udine), patients only underwent a medical interview and physical and gynaecological evaluations, without routine preoperative laboratory tests or X-rays.

**Table I:** Basic demographic characteristics of the patients, characteristics of the leiomyomas and operative outcomes (intraoperative, postoperative).

Variable		Centre A (Rome) N (%)	Centre B (Udine) N (%)
Type of surgery		Tot 1191	Tot 500
	Diagnostic Hysteroscopy + endometrial biopsy	119 (10%)	108 (21.6%)
	Polypectomy	654 (54.9%)	211 (42.2%)
	Myomectomy	136 (11.5%)	29 (5.8%)
	Blind D&C	5 (0.4%)	13 (2.6%)
	LEEP/conisation	223 (18.7%)	117 (23.4%)
	Vulvar procedures	6 (0.5%)	16 (3.2%)
	Uterine malformation (metroplasty)	48 (4%)	6 (1.2%)

The characteristics of each group are presented in Tables II and III. In the first group (Group A) the median age at intervention was 48.1 years (range 14-87) and the median BMI was 25.2 kg/ m2 (range 15-53). In the second group (Group B), the median age was 56.7 years (range 22-91), and the median BMI was 26.2 kg/m2 (range 15- 61). In both groups the median ASA score was 2 (indicating mild systemic disease). The median surgical time differed significantly between the two groups, with 24 minutes (range 1-80) for Group A and 19 minutes (range 2-80) for Group B (p=.0001). No significant differences were observed in perioperative and postoperative outcomes or time of discharge between the two groups. Specifically, we observed 14 (1.2%) and 7 (1.4%) postoperative complications in Groups A and B, respectively (p=0.70). Among these, 4 severe complications were reported in Group A (0.3%): two hospital readmissions for anaemia and blood transfusion and two readmissions requiring repeat surgery for uterine perforation with hemoperitoneum. In Group B, two cases of heavy bleeding (0.4%) occurred after conisation and cervical cancer biopsy, requiring reintervention for control of bleeding. Lastly, regarding cost reduction, in patients not subjected to routine blood sampling and chest X-rays (Group B), we observed a saving of approximately 70 Euros for each procedure (p < 0.001).

**Table II:** Population characteristics.

Variable		Group A (Roma)	Group B (Udine)	p value
Number of patients	1191	500	-
Age, year, median (range)	48.1 (14 – 87)	56.7 (22 – 91)	< 0.001
BMI (Kg/m2), Median (range)	25.2 (15 - 53)	26.2 (15 – 61)	0.002
ASA Median (range)	2 (1-2)	2 (1-2)	0.645

**Table III:** Surgical features and perioperative outcomes.

Variable		Group A (Rome)	Group B (Udine)	p value
All patients - N°	1191	500	-
Surgical Time - median (range); minutes	24 (1-80)	19 (2-80)	< 0.001
Hospital stay – median (range); days	0 (0-10)	0 (0-8)	0.59
Hospital readmission (%)	0.08%	0.20%	0.805
Surgical complications – N° (%)	14 (1.2%)	7 (1.4%)	0.70
Severe complications – N° (%)	4 (0.3%)	2 (0.4%)	0.84
Lab test costs for each procedure – (EUR)	55	0	< 0.001
Chest X-rays costs for each procedure – (EUR)	15	0	< 0.001

## Discussion

The term “minor gynaecological surgery” encompasses a broad range of surgical procedures that can typically be conducted within an outpatient model of care (Carugno et al., 2022). These procedures, characterised by short surgical time and minimal invasiveness, constitute the majority of surgical procedures performed annually in gynaecological facilities. Consequently, their impact on the healthcare system is significant. Debate persists across various specialities regarding the necessity of a comprehensive and standardised preoperative assessment. This study examines two distinct approaches to patient assessment, aiming to evaluate the potential impact of routinely conducted preoperative blood tests on perioperative outcomes. While population characteristics such as age and BMI slightly vary between the two groups, the ASA score did not show statistically significant differences. Group A exhibited a significantly longer median surgical time. This discrepancy may be attributed to a greater prevalence of complex procedures – such as advanced hysteroscopic interventions, challenging hysteroscopic myomectomies and surgeries for complex genital tract malformations – performed at a specialised referral centre like the CLASS Hysteroscopy Centre. The complication rate did not show statistically significant differences between the two groups, with predominantly mild complications such as bleeding or fluid overload syndrome, which were managed pharmacologically. Consistent with existing literature, six cases of severe postoperative complications were reported: four in Group A (0.3%), comprising two hospital readmissions due to heavy bleeding and anaemia necessitating blood transfusion, and two readmissions requiring repeat surgery for uterine perforation with hemoperitoneum or sepsis; and two in Group B (0.4%) both treated with reintervention due to persistent bleeding (Mutter et al., 2021). In a few cases, postoperative blood tests were conducted based on anaesthetic indication, to better assess clinical status, but none led to a change in management. In our population, clinically significant abnormalities in preoperative lab tests were found in less than 1% of cases, consistent with the research from Kaplan et al. (1985) In their study, abnormalities influencing perioperative management were reported in only 0.22% of cases, and essentially none of them resulted in adverse surgical or anaesthetic consequences. Another survey conducted in a general surgery setting reported that only 1.3% of routine tests performed before outpatient procedures were associated with clinically significant abnormalities and only half of these led to a change in the surgical plan (Wattsman and Davies, 1997). Similarly, in our population, preoperative blood assessment wouldn’t have altered management decisions as complications were primarily attributable to the surgical procedure itself. For example, excessive bleeding experienced by two patients in Group B (conisation and a cervical biopsy for cervical cancer, who did not receive preoperative examinations) was related to the underlying disease and cervical hypervascularisation. Additional lab tests conducted later for an in-depth analysis showed no coagulative alterations. These findings align also with literature reports that identify surgeon expertise and procedure type as the primary risk factors for complications in minor gynaecological surgery (Jansen et al., 2000). Regarding hysteroscopy, the most performed procedure in our study population, searching for studies on how to prevent perioperative complications, no mention is made of presurgical blood assessment. On the other hand, the literature emphasises the importance of preoperative ultrasonography for planning procedures, especially in cases of severe intrauterine adhesions, large myomas or uterine malformations (McGurgan and McIlwaine, 2015; Vilà Famada et al., 2022; Aas-Eng et al., 2017). There is growing evidence that sonographic imaging is increasingly pivotal in providing critical diagnostic information (Ricci et al., 2022). Moreover, in these delicate cases, the possibility of simultaneously using ultrasound and endoscopy to guide surgery, as in a Digital Hysteroscopic Clinic setting, may further reduce complication rates related to the surgical procedure itself (Campo et al., 2018).

In conclusion, consistent with the published literature, performing routine preoperative haematological and biochemical assessment for every patient undergoing minor gynaecological surgery did not reduce complication rates (Fleisher et al., 2014; Taylor et al., 2022). Additionally, no statistically significant differences were observed in overall postoperative complication rates, major complications, unplanned return to the theatre, hospital readmission or mortality. Moreover, considering the costs associated with preoperative assessments, a selective approach could yield a cost saving of at least 83,000 Euros, according to Lazio’s regional tariff schedule. To make a more general statement, despite the varying fees for healthcare services across different regions of Italy and thus the potentially significant differences in expenditure, it can still be asserted that each saving is statistically significant. Additionally, for a more comprehensive analysis, it’s essential to include the salaries of nurses and all other healthcare professionals involved in preoperatory assessment, leading to a significant increase in costs for the public health system. These considerations must be viewed within the broader context of rising health expenditure per capita and the need to avoid unnecessary public health costs. Additionally, in Italy health insurance coverage is limited, further exacerbating the burden on public finances. This study demonstrates that minor gynaecological surgery is a setting in which avoidance of superfluous testing can be implemented and there is still a long way to go to fully comply with guidelines. As proposed by the American Society of Anesthesiologists and many other scientific societies, selective preoperative testing is essential (Committee on Standards and Practice Parameters, 2012; NICE, 2016; Fleisher et al., 2014). A meticulous clinical evaluation by both surgeons and anaesthetists enables the identification of higher risk patients, for whom preoperative testing might reduce complications and improve outcomes. To our knowledge, this is the first study focused on cost-effectiveness. The inclusion of a wide variety of surgical procedures may have increased variability in both surgical times and complication rates between the two study populations. Another limitation of this interim analysis is the limited sample size as it may introduce statistical bias. Further analysis of the entire study population is necessary to validate these findings with adequate statistical power.

## Conclusions

This preliminary analysis indicates that routine preoperative testing does not correlate with improved outcomes in terms of postoperative complications in patients undergoing minor gynaecological surgery. Additionally, our sample did not show statistically significant differences in major complications, hospital readmissions or surgical reinterventions. Moreover, considering the volume of procedures analysed, a reduction in routine preoperative expenditure results in a considerable cost reduction. This aspect can’t be disregarded in the implementation of a modern healthcare policy. A joint statement issued from both gynaecological and anaesthetic societies could establish a unified guideline for the safest management of these patients
